# Histone Deacetylases in the Inflamed Intestinal Epithelium—Promises of New Therapeutic Strategies

**DOI:** 10.3389/fmed.2021.655956

**Published:** 2021-03-26

**Authors:** Lorenz Gerbeth, Rainer Glauben

**Affiliations:** ^1^Department of Gastroenterology, Infectious Diseases and Rheumatology, Charité - Universitätsmedizin Berlin, Corporate Member of Freie Universität Berlin and Humboldt-Universität zu Berlin, Berlin, Germany; ^2^Department of Medical Biotechnology, Institute of Biotechnology, Technische Universität Berlin, Berlin, Germany

**Keywords:** histone deacetylase, HDAC, inflammatory bowel disease, intestinal epithelium, HDAC inhibitor, inflammation

## Abstract

The intestinal epithelium is a complex, dynamic barrier that separates luminal contents from the immune compartment while mediating nutrient absorption and controlled passage of antigens to convey oral tolerance. A compromised epithelial barrier often leads to inflammation because immune cells in the lamina propria come into direct contact with luminal antigens. Defects in epithelial cell function were also shown to be involved in the etiology of inflammatory bowel diseases. These are severe, chronically relapsing inflammatory conditions of the gastrointestinal tract that also increase the risk of developing colorectal cancer. Despite major efforts of the scientific community, the precise causes and drivers of these conditions still remain largely obscured impeding the development of a permanent cure. Current therapeutic approaches mostly focus on alleviating symptoms by targeting immune cell signaling. The protein family of histone deacetylases (HDACs) has gained increasing attention over the last years, as HDAC inhibitors were shown to be potent tumor cell suppressors and also alleviate morbid inflammatory responses. Recent research continuously identifies new roles for specific HDACs suggesting that HDACs influence the cell signaling network from many different angles. This makes HDACs very interesting targets for therapeutic approaches but predicting effects after system manipulations can be difficult. In this review, we want to provide a comprehensive overview of current knowledge about the individual roles of HDACs in the intestinal epithelium to evaluate their therapeutic potential for inflammatory conditions of the gut.

## Introduction

The intestinal epithelium is a highly dynamic tissue whose functional integrity is indispensable for proper gut homeostasis. Lining the inner walls of the gastrointestinal tract, it establishes the first line of defense from potential pathogens contained in ingested material but simultaneously allows controlled passage of nutrients and selected antigens. Functional defects of the intestinal epithelium can lead to severe dysregulations of gut homeostasis and are a hallmark of many chronic gastrointestinal conditions such as inflammatory bowel diseases (IBD) (1). IBD, with Crohn's disease (CD) and ulcerative colitis (UC) being the most frequent forms, is characterized by chronically relapsing, exaggerated inflammation that involves drastic alterations in the microbiome and epithelial barrier function (2, 3). Despite rising incidence rates worldwide and extensive research, the precise etiology and drivers of IBD are still not clear with only few therapeutic options and no permanent cure available (4).

The promise of a new therapeutic approach arose when we could show that SAHA (Vorinostat), an inhibitor of histone deacetylases (HDACs), has the potential to alleviate intestinal inflammation in an IBD mouse model (5). Previously, HDAC inhibitors were mainly appreciated for their anticancer activity (6). At the time, most small molecules used in these studies were pan-HDAC inhibitors, meaning they inhibit all members of the classical HDAC family (7). In the mammalian genome, this protein family comprises 11 HDACs, that, are subdivided into three classes depending on their structure, enzymatic function, subcellular localization, and expression patterns (Table 1) (22). The eponymous function of epigenetic control via histone deacetylation is mainly implemented by class I HDACs while members of the other classes have either mainly non-histone targets or display a strongly reduced catalytic activity in their deacetylation domain and are considered to function rather *via* sequestering their targets than deacetylating them (22). HDACs are a phylogenetically very old protein family and are deeply rooted into the cellular signaling network. Therapeutic strategies that base on inhibiting all members of this family could therefore bear a certain disruptive potential, which might not be directly evident.

**Table 1:** Superfamily of Zn2+-dependent histone deacetylases (HDACs) of the mammalian genome with subcellular localization and reported role in intestinal epithelial cells (IECs) during inflammation.

	**HDAC**	**Subcellular localization**	**Reported role in IECs during inflammation**	**References**
Class I	HDAC1	Nuclear	Negative regulator of STAT signaling, NF-κB signaling and acute phase response; involved in IL-1β-dependent cytokine production; positive regulator of the p38/MAPK pathway; downregulation of tight-junction proteins	(8–11)
	HDAC2	Nuclear	Positive regulator of inflammatory response and serotonin transporter; negative regulator of STAT signaling and expression of antibacterial lectins	(10, 12, 13)
	HDAC3	Nuclear and cytoplasmic	Negative regulator of retinoic acid metabolism and NF-κB signaling, deacetylation of p65; downregulation of tight-junction proteins; crosstalk with microbiome; activation of intraepithelial lymphocytes during infection	(11, 14–17)
	HDAC8	Nuclear and cytoplasmic	NA	
Class IIa	HDAC4	Nuclear and cytoplasmic	Invovled in acute phase response, interacts with C/EBPδ	(18)
	HDAC5	Nuclear and cytoplasmic	NA	
	HDAC7	Nuclear and cytoplasmic	NA	
	HDAC9	Nuclear and cytoplasmic	NA	
Class IIb	HDAC6	Nuclear and cytoplasmic	Positive regulator of NF-κB signaling; downregulation of tight-junction proteins	(19, 20)
	HDAC10	Nuclear and cytoplasmic	NA	
Class IV	HDAC11	Nuclear and cytoplasmic	Downregulation of tight-junction proteins in response to LPS	(21)

The environment of the intestinal mucosa adds an additional level of complexity to this issue, as different cell types are involved whose signaling network might rely on different HDACs with different functions. Many studies have looked into the role of HDACs in immune cells as the obvious mediators of inflammation (23). As increasing evidence over the last years also ascribed crucial immune regulatory functions to the intestinal epithelium, many recent studies also reported on the role of HDACs in the intestinal epithelium during inflammation. Here, we want to condense their results to provide a bigger picture about currently known inflammation-associated signaling pathways that involve HDAC signaling in intestinal epithelial cells (IECs) to help improve our understanding of the effects of HDAC inhibitor treatment on the intestinal epithelium. Additionally, to explore possibilities of a more targeted treatment, we outline the current knowledge about the roles of specific single HDACs in this context.

### Pan-HDAC Inhibition

In the gut, HDAC inhibition is a naturally occurring mechanism that constitutes an integral part of homeostasis. Short chain fatty acids (SCFAs), such as butyrate, propionate, and acetate, produced by various bacterial communities of the microbiome mostly *via* anaerobic fermentation of dietary fibers, act as natural HDAC inhibitors (24, 25). In particular, besides serving as energy source for colonocytes, butyrate elicits a wide array of beneficial effects for gut homeostasis including suppression of pathological inflammation (26).

Considerable advances in understanding the importance of HDACs for inflammatory response pathways in IECs have been made by investigating the anti-inflammatory properties of butyrate and other SCFAs. The influence of butyrate on cell signaling can often be traced back to its ability to act as an HDAC inhibitor. For example, butyrate was found to support barrier function by increasing expression of IL-10 receptor α subunit (IL-10RA) via activation of STAT3 in human colon-derived cell lines Caco-2 and T84. In turn, IL-10RA increases tightness of the epithelial barrier by mediating downregulation of the pore-forming claudin-2. This mechanism depends on HDAC activity, as it can be reproduced by other pan-HDAC inhibitors, such as Trichostatin A (TSA) (27). Similarly, using cell lines and enteroids from mouse and human, the conductive effects of butyrate on the production of retinoic acid, an important immune regulator, could be ascribed to HDAC inhibition in IECs (17).

HDACs are most likely also the main mediators for conveying the effects of butyrate and propionate on nuclear factor kappa light-chain-enhancer of activated B-cells (NF-κB) signaling in response to Toll-like receptor (TLR) or TNFα stimulation. In cell culture models of human colon IECs, HDAC inhibition by butyrate and propionate increase TNFα and decrease IL-8 and MCP-1 expression in response to TLR5 stimulation (28). By contrast, during steady state, phenyl butyrate increases IL-8 and IL-18 production as well as TLR2-dependent expression of host defense peptides pEP2C, pBD-1, and pBD-3 in porcine IECs (29). HDAC inhibition by TSA dramatically increases the production of antimicrobial peptides, such as β-defensins, upon bacterial challenge in cell lines and organoids of human colon epithelium (30). TSA induces phosphorylation of the IκB kinase complex, which in turn phosphorylates inhibitor of NF-κB alpha (IκBα) and serine 10 of histone H3 activating NF-κB signaling and expression of target genes, respectively (30). Silencing of TLR2 or TLR4 increases overall HDAC activity and considerably mitigates the effects of phenyl butyrate on host defense peptide expression (29). Interestingly, TLR2 and TLR4 are two of the main receptors for recognizing extracellular high-mobility group box 1 (HMGB1), which plays an important role in the pathogenesis of IBD and whose secretion is also controlled by HDAC activity (31, 32). HMGB1 is typically localized in the nucleus but can be released into the extracellular space upon stress or tissue damage acting as a damage-associated molecular pattern (DAMP) that induces pro-inflammatory responses by binding its receptors (33). In a study investigating the anti-inflammatory effects of flavonoid isoliquirtigenin using HT-29 cells (human colon IECs), isoliquirtigenin prevented HMGB1 acetylation, leading to subsequent cytosolic translocation and secretion, by increasing HDAC activity (32). While HDACs appear to be negative regulators of TLR2 and TLR4, signal transduction of the intracellular virus-sensing receptor TLR3 heavily depends on HDAC activity. The HDAC inhibitor SAHA causes strong downregulation of TLR3 supposedly through upregulation of interferon response factor 8, which suppresses TLR3 transcription (34). Consequently, SAHA-treated IECs do not react to TLR3 stimulants with upregulation of TLR3-responsive target genes, such as IL-6, TNFα, and IFNβ, or phosphorylation and activation of NF-κB and MAP kinases ERK and JNK (34). In contrast, antiviral defense mechanisms involving IFN-responsive gene induction in response to type III interferons are significantly increased in murine IECs when HDAC activity is hampered (35). The differential influence of HDAC activity on TLR signaling demonstrates the complexity by which HDACs affect certain cellular responses.

We showed recently that the pan-HDAC inhibitors SAHA and ITF2357 (Givinostat) protect the epithelial barrier integrity from TNFα-induced disruption by upregulating expression of tight junction proteins occludin and claudin-1 while downregulating claudin-2 in *in vitro* monolayer models of T84 and CMT93 (murine IECs) cells. HDAC inhibition further supports wound healing by upregulation of IL-8 and TGFβ during inflammation in cell lines and primary murine enteroids. Oral administration of ITF2357 significantly improves regeneration after acute DSS-colitis and alleviates symptoms of inflammation in mice (36). Another recent study linked the beneficial effects of HDAC inhibition in the intestinal epithelium during inflammation to expression changes of the IL-12 cytokine family (37). The heterodimeric members of this protein family can convey inflammatory or anti-inflammatory effects depending on their subunits and play important roles in intestinal inflammation (38). One of these subunits, Epstein-Barr virus-induced gene 3 (EBI3), becomes highly upregulated in human colon epithelial cells when TNFα treatment is combined with HDAC inhibition *via* Trichostatin A (TSA). Considering expression levels of other IL-12 subunits and activated signaling pathways in the cell, the authors suggest that the anti-inflammatory properties of HDAC inhibition in the intestinal epithelium are mainly conveyed through increased formation of the anti-inflammatory IL-35, which is also upregulated in acute phases of ulcerative colitis (37, 39). Strikingly, the anti-inflammatory effects of HDAC inhibition in a DSS-colitis mouse model are completely abolished and even reversed into exacerbation of the disease phenotype when *Ebi3* is silenced indicating a crucial role of EBI3 in mediating the beneficial effects of HDAC inhibition in intestinal inflammation (37).

Research with SCFAs and chemical pan-HDAC inhibitors demonstrates that HDACs play an important role in multiple inflammation-associated pathways in the intestinal epithelium. Yet, potentially distinct roles of single HDACs can only be inferred to a very limited degree from this data. However, considering HDACs as therapeutic targets, pan-inhibition might be neither a necessary nor the safest option. Targeting only single HDACs is likely more efficient and limits undesired off-target effects. The field of HDAC research is still relatively young and the tissue specific expression patterns and functions make their study even more challenging. Nevertheless, many recent studies provided new insights into the functions of single HDACs in the IECs.

### Class I HDACs

Class I HDACs are the most intensively studied group of this protein family and are often considered the “true” HDACs since they exert epigenetic control through deacetylation activity toward histones. In rat IEC-6 cells, the class I HDAC HDAC1 was shown to control global acetylation levels (40). Alterations in activity of class I HDACs often lead to profound, global changes of histone acetylation patterns and associated gene expression. In active sites of UC or CD, IECs exhibit significantly decreased levels of histone H3 acetylation compared to healthy controls suggesting increased HDAC activity (11, 41). Indeed, HDAC activity also increases measurably in the inflamed colonic epithelium of mice treated with DSS (41). Paradoxically, most HDAC transcripts, including the class I HDACs HDAC2, HDAC3, and HDAC8, are downregulated in the epithelium of active IBD patients (36). HDAC1 mRNA levels do not change during inflammation indicating a special role (36).

Indeed, HDAC1 was reported to be an important regulator of inflammatory responses in IECs but also to be involved in certain aspects of homeostasis. Silencing of *HDAC1* impairs cell proliferation and alters cell morphology of rat IECs (8). These effects are most likely an indirect consequence of metabolic reprogramming including downregulation of homeostatic processes and upregulation of survival pathways (40). The cells produce less ATP but are more resistant to nutrient deficiency and oxidative stress (40). In terms of inflammation, HDAC1 depletion causes prolonged activity of the acute phase response and NF-κB signaling by retention of phosphorylated C/EBPβ and phosphorylated p65 in the nucleus upon IL-1β stimulation (8). Interestingly, HDAC1 silencing causes elevated levels of certain inflammatory cytokines in response to IL-1β, such as Cx3cl1, Timp1, and Cxcl2, while others are decreased, such as Cxcl5 and β-NGF (8). *In vitro*, HDAC1 becomes upregulated in human IECs when stimulated with IL-4, IL-5, IL-13, MCP-1, or TNFα, all being activators of the p38/MAPK pathway (9).

HDAC2 is in many ways closely associated to HDAC1 signaling. Certain DNA-binding multiprotein complexes, such as Sin3A, NuRD, or CoREST, require incorporation of HDAC1 and HDAC2 as heterodimer to exert their biological activity and HDAC2 protein levels increase after *Hdac1* silencing suggesting some form of substitution (8, 42). Epithelial HDAC1 and HDAC2 are of critical importance for intestinal homeostasis as simultaneous deletion of both genes in IECs of adult mice leads to profound dysregulations across multiple cell signaling pathways (43). This involves altered tissue architecture caused by an increased proliferative and migratory activity of IECs, differentiation defects affecting especially secretory lineages leading to decreased numbers of goblet cells and Paneth cells, and increased expression of inflammation-associated genes inflicting weight loss and colon shortening (43). Mechanistically, these effects were traced back to changes in the expression levels of certain key regulators. Increased expression of Cyclin D and targets of the mTOR pathway affect cell proliferation and division while elevated levels of activated Notch shift cell fate determination from a secretory to an absorptive phenotype (43). In addition, IEC-specific *Hdac1/2* deletion decreases expression levels of tight junction protein claudin-3 thereby weakening the intestinal barrier and leading to activation of inflammatory regulators, such as Stat3 (43). Combined with reduced microbial protection due to decreased secretion of mucus and antimicrobial products from a diminished number of secretory cells, the tissue exhibits a phenotype of basal chronic inflammation with increased immune cell infiltration (43). Accordingly, IEC-specific *Hdac1/2* knockout mice suffer considerably aggravated symptoms when subjected to DSS-induced colitis (12). Interestingly, IEC-specific deletion of *Hdac2* alone appears to protect mutant mice from DSS colitis as they lose less weight and retain a higher epithelial barrier integrity compared to wild type mice (12). Immune programs are strongly downregulated in these mice while antibacterial lectins, such as Reg3b and Reg3g are strongly increased (12). Silencing of *HDAC2* in Caco-2 cells additionally decreases expression of the transporter of serotonin, whose expression is commonly dysregulated in inflammatory bowel disease (13). Comprehensive analysis of murine intestinal organoids with a *Hdac1* or *Hdac2* deletion suggests that HDAC2 influences the intestinal immune response and regulation of the intestinal barrier function through its involvement in xenobiotic signaling and the aryl hydrocarbon receptor-mediated response to endogenous and exogenous ligands (10). STAT signaling is increased after *Hdac1* or *Hdac2* knockout suggesting them as negative regulators for this pathway (10).

HDAC3 is important for a variety of epithelial cell functions particularly concerning cross-talk with the microbiome. Mice with an IEC-specific *Hdac3* knockout are more susceptible to DSS-induced inflammation and intestinal damage (14). This phenotype may in part be caused by increased activation of NF-κB. HDAC3 was previously shown to restrict NF-κB activity by deacetylating p65 promoting its nuclear export and binding to IκBα (15). Therefore, HDAC3 might be the main mediator for the reported activating effect of phenyl butyrate on NF-κB signaling (29). IEC-specific *Hdac3* knockout mice also display loss of Paneth cells, impaired IEC function, decreased expression of antimicrobial peptides, and altered composition of commensal bacteria (14). Interestingly, this phenotype can be rescued by transferring the animals to germ-free conditions suggesting that HDAC3 is necessary for integrating signals from the microbiome during homeostasis (14). IEC-intrinsic HDAC3 has also been shown to regulate activation of IFNγ-producing intraepithelial lymphocytes by inducing IL-18 expression in the epithelium upon bacterial infection (16).

The class I HDACs HDAC1, HDAC2, and HDAC3 are evidently involved in the regulation of the inflammatory response in IECs. However, describing a precise mechanism of action is still challenging. The available data raise the possibility that the anti-inflammatory properties of pan-HDAC inhibitors are mostly mediated through inhibition of class I HDACs. Inhibition of additional members of the HDAC family might not add to the desired result unnecessarily increasing the risk of off-target effects. For example, the effects of butyrate on STAT signaling and retinoic acid metabolism (see above) might mainly be due to decreased HDAC1 and HDAC2 activity as silencing either is sufficient to reproduce this effect (10, 17). Indeed, symptoms of intestinal inflammation in a DSS-colitis model can also be alleviated with more specific inhibitors, such as MS-275 (Entinostat) that inhibits mainly HDAC1 and HDAC3 activity (11, 44). Inflammation-induced reduction of acetylation, activation of NF-κB, and downregulation of tight-junction proteins zonula occludens 1 (ZO-1) and occludin are all reversed by MS-275 treatment (11). The enhancing effect of pan-HDAC inhibition on IFN-responsive gene induction in response to type III interferons in murine IECs can also be reproduced by inhibiting HDAC1 and HDAC3 alone *via* MS-275 (35). Class I HDACs could drive inflammation by controlling expression of certain key regulators, such as the vitamin D receptor (11), but also by deacetylating proteins of the inflammation signaling chain, such as p65, thereby affecting their activity (15).

### Class II HDACs

HDACs of class II are further subdivided into class IIa, containing HDAC4, HDAC5, HDAC7, and HDAC9, and class IIb, containing HDAC6 and HDAC10. Class IIa HDACs influence gene expression by interacting with various transcription factors mostly suppressing their activity. Conserved residues in the protein sequence of class IIa HDACs can be phosphorylated triggering nuclear export (22). To date, the roles of most class II HDACs in the intestinal epithelium are only scarcely investigated. Class IIa HDACs were described as crucial components of protein kinase D1 (PKD1)-dependent mitogenic signaling (45). HDAC4 might play a role in the acute phase response during inflammation as it interacts with C/EBPδ, a key regulator of haptoglobin expression, in cultured IEC models (18). Epithelial HDAC7 was found to be positively associated with development of colorectal cancer (46).

HDAC6 represents a very interesting therapeutic target in intestinal inflammation, as it was recently shown to be important for NF-κB signaling. In a human colonic cell line, the HDAC6-specific inhibitor CKD-506 blocks phosphorylation of IκBα, suppresses IL-8 secretion, and inhibits DNA binding of the NF-κB complex (20). In mouse models of experimental colitis, oral administration of CKD-506 significantly improves symptoms of intestinal inflammation (20). A similarly beneficial effect of HDAC6 inhibition has been found in the context of reperfusion damage of the intestine after hemorrhagic shock (HS). Inhibition of HDAC6 *via* Tubastatin-A prevents loss of tight junction proteins claudin-3 and ZO-1 and attenuates injury-induced tissue alterations, such as villous blunting, epithelial necrosis, and immune cell infiltration in a murine HS model (19).

### Class IV HDAC

The only class IV HDAC, HDAC11, has been suggested to play a role in LPS-induced downregulation of tight-junction proteins and subsequent loss of barrier integrity. In human intestinal epithelial cells, Vitamin D protects LPS-induced loss of barrier integrity by upregulation of its receptor, which sequesters HDAC11 and prevents its recruitment to the DNA (21). Chromatin immunoprecipitation revealed ZO-1, claudin-5, and occludin as targets of HDAC11, which binds to their promoters and impairs gene transcription in response to LPS stimulation (21).

## Concluding Remarks

An increasing number of independent studies show that HDACs influence inflammation and barrier function in the intestinal epithelium. Therefore, epithelial HDACs definitely represent promising therapeutic targets that could help to control inflammation and protect barrier integrity in diseases like IBD. HDACs are involved in many inflammatory signaling pathways *via* direct interaction with key regulators or influencing their gene expression. Although many mechanisms of action have already been identified for single HDACs, drawing a comprehensive picture still proves challenging. Especially class I HDACs affect a large number of cellular responses due to their epigenetic activity and extensive effects on gene expression. Data on the role of class II and IV HDACs in IECs is still very limited but they could represent more specific therapeutic targets since they do not affect global histone acetylation levels to the same extend as class I HDACs. This, however, remains to be clarified by future studies.

A major weak point of current HDAC research is that many studies that report a certain role for HDACs in IECs rarely focus on single HDACs or even have the function of HDACs as a primary study goal. HDACs often appear as a side note, a secondary finding that happened to be connected to the initial point of interest. Further elaborations on the precise underlying modes of action that integrate specific HDACs into the signaling pathway under investigation are often missing. However, the fact that HDACs appear in so many different contexts, especially with a focus on inflammation, shows the enormous potential that lies within detailed knowledge of their individual roles. Future studies, which aim directly at deciphering the role of specific HDACs in distinct cell types, are necessary to build on current knowledge and enable novel therapeutic strategies for IBD and other inflammatory diseases by precise modulation of HDAC activity.

## Author Contributions

LG and RG organized the review structure. LG performed the bibliographic research and wrote the manuscript. Both authors were involved in editing the paper and had final approval of the submitted version.

## Conflict of Interest

The authors declare that the research was conducted in the absence of any commercial or financial relationships that could be construed as a potential conflict of interest.
